# Author Correction: Safety and immunogenicity of a tetravalent and bivalent SARS-CoV-2 protein booster vaccine in men

**DOI:** 10.1038/s41467-024-44729-x

**Published:** 2024-01-08

**Authors:** Suad Hannawi, Linda Saf Eldin, Alaa Abuquta, Ahmad Alamadi, Sally A. Mahmoud, Aala Hassan, Shuping Xu, Jian Li, Dongfang Liu, Adam Abdul Hakeem Baidoo, Dima Ibrahim, Mojtaba Alhaj, Yuanxin Chen, Qiang Zhou, Liangzhi Xie

**Affiliations:** 1 Internal Medicine Department, Al Kuwait-Dubai (ALBaraha) Hospital, Dubai, United Arab Emirates; 2 General Surgery Department, Al Kuwait-Dubai (ALBaraha) Hospital, Dubai, United Arab Emirates; 3 Ear, Nose and Throat Department (ENT), Al Kuwait-Dubai (ALBaraha) Hospital, Dubai, United Arab Emirates; 4 Biogenix labs, G42 Healthcare, Dubai, United Arab Emirates; 5 Beijing Engineering Research Center of Protein and Antibody, Sinocelltech Ltd., Beijing, China; 6 Infectious Diseases Department, Burjeel Medical City, Abu Dhabi, United Arab Emirates; 7 Research Department, Burjeel Medical City, Abu Dhabi, United Arab Emirates; 8 Cell Culture Engineering Center, Chinese Academy of Medical Sciences & Peking Union Medical College, Beijing, China

**Keywords:** Viral infection, Drug development

Correction to: *Nature Communications* 10.1038/s41467-023-39766-x, published online 08 July 2023

In this article, the author name Linda Saf Eldin was incorrectly written as Linda Saifeldin. The original article has been corrected.

